# Variables Associated with Attitudes toward Biodebridement Using *Lucilia sericata* Larvae in a Group of Nurses

**DOI:** 10.3390/healthcare11233081

**Published:** 2023-12-01

**Authors:** Dariusz Bazaliński, Kamila Pytlak, Joanna Przybek-Mita, Paulina Szymańska, Anna Wójcik, Aneta Zymon, Ronald Sherman, Albert Nguyen, Izabela Sałacińska, Paweł Więch

**Affiliations:** 1 Institute of Health Sciences, College of Medical Sciences, University of Rzeszów, 35-959 Rzeszów, Poland; dbazalinski@ur.edu.pl (D.B.); joprzybek@ur.edu.pl (J.P.-M.); wojcik95@poczta.fm (A.W.); isalacinska@ur.edu.pl (I.S.); 2 Podkarpackie Specialist Oncology Centre, Specialist Hospital in Brzozów, 36-200 Brzozów, Poland; kamila.pytlak@interia.pl; 3 Postgraduate Nursing and Midwifery Education Centre, 35-083 Rzeszów, Poland; 4 Department of Vascular Surgery, Specialist Hospital in Radom, 20-617 Radom, Poland; mobilnapielegniarka@gmail.com; 5 Ultramed—Center for the Treatment of Vascular Diseases, Wounds and Pain, 30-002 Krakow, Poland; aneta@zymon.net; 6 BioTherapeutics, Education and Research (BTER) Foundation, Irvine, CA 92617, USA; rsherman@uci.edu (R.S.); albertbnguyen9@gmail.com (A.N.)

**Keywords:** nurse, gender, chronic wound, perception, debridement, *Lucilia sericata*

## Abstract

Despite numerous studies and recommendations, the acceptance of treatments involving medicinal maggots in many clinics has been slow. Several factors may account for this, including the gender of nurses administering the treatment, their level of work experience, and their perceived level of personal stress. The aim of the study was to assess the impact of selected variables (gender, work experience, stress level) on the readiness of nurses to administer maggot debridement therapy (MDT), which is a form of biodebridement. The study population was a cohort of 290 wound care nurses providing specialist care for patients with chronic wounds. It was assumed that the identified variables may determine the implementation of larval therapy in everyday professional practice. A subsample of 35 men and 35 women was further analyzed to determine if gender, work experience, and/or personal stress levels were correlated with attitudes towards the utilization of maggots in biodebridement. Assessment tools included the Perceived Stress Scale (PSS-10) and the MDT 10 Perception Assessment Questionnaire, a protocol by which the subject ranked six wound photographs in order of repulsiveness and responded to questions regarding demographic variables, which include education and work experience. The visual perception of pictures of a wound with larvae is indirectly an indicator of the attitude towards larval therapy. Selection of the photograph with maggots on the wound as the most repulsive image was associated with a personal appraisal of not being ready to implement maggot therapy (chi-square = 8.430, *p* = 0.015). Low work experience (chi-square = 14.039, df = 4, *p* = 0.007), and low readiness for MDT (chi-square = 8.430, df = 2, *p* = 0.015) were also associated with unpreparedness to administer maggot therapy. Neither gender nor perceived stress level were exclusively associated with disgust for maggots or lack of readiness to implement MDT. Low professional experience and a deficit of knowledge in maggot therapy may negatively affect the readiness of nurses to administer biodebridement. Gender and personal stress levels do not affect nurses’ readiness to utilize larval therapy.

## 1. Introduction

Wound debridement is a crucial local activity that is necessary to eliminate infected necrotic tissue from the wound bed. Debridement stimulates repair processes and the growth of granulation tissue, reduces the risk of infection, and eliminates odor and exudates [1]. Debridement methods include surgical, acute (nursing with sharp instruments), enzymatic, autolytic, and biological interventions [2,3]. The benefits of maggots have been known for centuries, but in recent years the popularity of the maggot wound dressings method, referred to as maggot debridement therapy (MDT) or biosurgery, has significantly increased in its standing and use and is no longer considered solely as an alternative or “last chance” method. This therapy utilizes live larvae of the green bottle fly (most commonly *Lucilia sericata*), which are reared in controlled and sterile laboratory conditions. The use of MDT in the wound healing process not only debrides the wound of necrotic tissue, but also eliminates biofilm and bacteria and stimulates repair processes in the wound. One of the main challenges associated with the elimination of biofilm is the increased resistance to antibiotics. A meta-analysis by Malone et al. confirms the presence of biofilm in 78.2% of chronic wounds [4]. Antibiotic therapy used without clinical justification contributes to the development of resistant microorganisms, thus allowing the development of multidrug-resistant strains (MDROs) [5,6].

According to wound care recommendations, the elimination of devitalized, necrotic tissue is a fundamental procedure in wound management [2,7,8]. Necrotic tissue and debris inhibit natural wound regenerative processes. There is no single best method for wound debridement. The method of necrotic tissue elimination must be selected according to a multitude of factors including the anatomical area, the location and depth of damaged structures, the amount of exudates, the severity of wound pain, the underlying medical conditions of the patient, and the patient’s preferences [9,10]. Mechanical wound debridement (rubbing, scraping, plucking, cutting) is the simplest, fastest, and often least expensive method of biofilm elimination performed by trained medical personnel [2,11,12,13]. Biodebridement (MDT) meets the criteria for both mechanical and autolytic methods due to excretions and secretions (ES) produced by the larvae. Studies conducted in recent years indicate a number of neoangiogenic factors, including those that activate endothelial cells. Indications for MDT are wounds of established etiology that are infected with the presence of devitalized or necrotic tissue in the course of conditions such as: diabetic foot syndrome, lower leg venous ulcers, mixed and arterial etiology (after previous revascularization treatment), pressure injuries, and other wounds requiring precise debridement and stimulation of healing after prior examination and evaluation of the patient’s condition. Key contraindications of MDT include dry and undiagnosed necrosis, atypical wounds, wounds with a neoplastic background, allergies to hittin, and lack of patient consent and acceptance of the treatment method [8,11,13].

The analysis of wounds before and after the application of medical larvae proves the promotion of wound healing on many levels. The appealing element of MDT is its broad, proteolytic effect from the secretion and excretion of specific enzymes and their correlation with antibacterial, antibiofilm, and anti-inflammatory activity; synergism with selected antibiotics; immunomodulating functions; and growth inhibition of selected cancers [8,10,12].

Experts from the Polish Wound Management Society (PTLR) recommend MDT for the treatment of full-thickness pressure injuries and infected diabetic foot ulcers [11,13].

Despite many studies demonstrating its efficacy and safety as well as recommendations from wound care experts and organizations [12,14,15], MDT remains scarce in clinical practice. There are several factors that may contribute to the reluctance of staff (doctors, nurses) and patients to accept maggot debridement therapy. One factor is the so-called “yuck factor”. Even though professional wound care providers are accustomed to the sight and odor of necrotic tissue, the thought of encountering wriggling larvae in those wounds might be more challenging for some people to comfortably bear. As a result, the response to the mere sight of images of larvae and disintegrating necrotic tissue may be a good predictor of disgust and reluctance to implement MDT. The unpleasant smell of necrotic wounds and the sight of the larvae may evoke negative experiences, especially in women [10,14]. For patients, concerns about the larvae escaping the wound dressing and crawling on their body could be additional factors that cause their revulsion towards MDT [16,17].

Advanced medical and nursing education can improve knowledge, skills, and personal attitudes. Being sensitive and empathetic to the patient’s needs, offering emotional and physical availability for the patient, and expressing commitment to high quality medical care are factors mainly connected to a nurse’s personality and the type of work environment. The work of an advanced practice nurse is complex and includes therapeutic decision making and independent patient care under a variety of conditions, both inside and outside of the hospital [18]. Despite the highly developed skills and experience of advanced wound care nurses, many of their duties and daily exposures are substantial stressors: caring for patients with traumatic body injuries, secretions, excretions, chronic and cancerous wounds, the smell of rotting tissue, pain, and many other experiences of human suffering [19]. A nurse’s skill of overcoming excessive emotional tension in difficult situations can affect their personal attitude, receptivity towards receiving more responsibilities, and their ability to deliver compassionate care. The literature indicates that work-related stress can come from both professional issues as well as suboptimal working conditions [17,20]. It is reasonable to assume that the level of perceived stress, combined with a deficit of knowledge about MDT, may reduce the potential readiness to use medical maggots in therapeutic protocols. The demographics and work experience of medical personnel may also affect one’s attitudes towards using MDT in clinical practice. It has already been shown that increased knowledge about MDT is associated with increased readiness to implement maggot therapy in the clinical setting [21]. During an interdisciplinary discussion of international cooperation and exchange of experience between therapists at the University of Rzeszów and the BioTherapeutics, Education, & Research (BTER) Foundation, Irvine, California, USA, an attempt was made to answer the question of what factors may determine a therapist’s reluctance to larval therapy, and what actions should be implemented in order to popularize the method [22]. The aim of the following study was to determine whether variables such as gender, professional experience, and personal stress level of the therapist affect the readiness associated with the use of *Lucilia sericata* larvae in a subgroup of previously selected pairs of nurses specializing in the prevention and treatment of wounds [16,21].

## 2. Materials and Methods

### 2.1. Ethics

The study protocol was approved by the ethics committees of the involved institution (Bioethics Commission at the University of Rzeszow: resolution No. 2018/01/07f on 7 January 2018). In addition, the principles of the Declaration of Helsinki were observed throughout the course of the study. Participants were informed about the purpose of the study and their right to withdraw at any point, for any reason.

### 2.2. Subjects

The main study, in accordance with the applicable methodological requirements, was conducted on a group of nurses practicing prophylaxis and wound treatment in out-of-hospital conditions. The study was voluntary and the purpose and structure of the questionnaire were discussed with the assigned investigator of the research team. Out of the entire group, 1136 people participated in training which was organized in 2020–2021 at the Postgraduate Education Center for Nurses and Midwives in Rzeszów, Poland. For the purposes of this study, the main sample of 290 people consisted of 255 women and 35 men. The majority of the sample were women (87.9%). The mean age was over 40 years; the median was 44 years. The median of work experience was 18 years [16,21]. All 35 men (100%) were qualified and divided into 6 categories, and then a randomization algorithm using a pseudo-random number generator in Excel (Microsoft, Redmond, WA, USA) was used. A group of 35 women in similar categories was randomized to a group of 35 men matched for ages. A sample of 70 people was created in this manner and then subjected to statistical analysis based on the adopted research assumptions.

### 2.3. Research Tool

**Figure 1:**
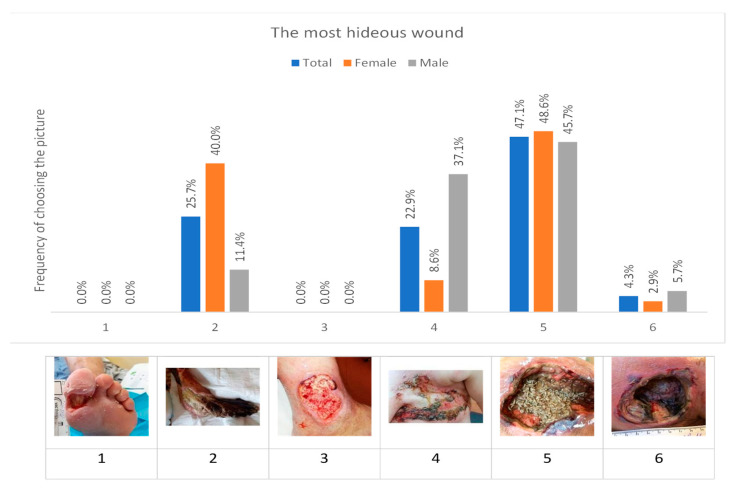
Selection of the most hideous photo of the wound in the opinion of the respondents.

The original study utilized a quantitative survey design consisting of four components, two of which include: (a) a questionnaire about the respondent’s sociodemographics, work experience, and education; (b) the five-point Likert scale “MDT 10 Perception Assessment”, developed by Bazaliński and Krawiec [21], consisting of 10 questions divided into two subscales (knowledge, motivation). Answers to questions were constructed on a five-point Likert scale (I strongly disagree, I rather disagree, I have no opinion, I rather agree, I strongly agree). For each positive answer, the respondent scored 5 points, with a total minimum of 10 points and maximum of 50 points. The higher the score, the greater the level of readiness to implement MDT. The reliability of the questionnaire on the perception of the use of MDT was determined at the Cronbach’s alpha level of α = 0.76. The third component of the quantitative survey design is the perceived stress scale (PSS-10), developed by S. Cohen et al. [23] and adapted to Polish by Ogińska-Bulik and Juczyński, containing 10 questions regarding various subjective feelings related to personal problems and events as well as behaviors and coping mechanisms. The results in the PSS-10 questionnaire range from 0 to 40 points. The higher the score, the higher the level of stress experienced. This tool helps determine the level of stress that an individual experienced one month before the test [24]. The fourth component of the quantitative survey design is a research protocol consisting of 6 photos of chronic wounds (Figure 1) of various etiologies selected at random from a prepared bank of 30 photos. The first photo (1) shows a diabetic foot with a hallux wound. The wound is debrided and prepared for treatment. The second photo (2) shows a necrotic foot in a geriatric patient at the end of life. The third photo (3) displays an ulceration of arteriovenous origin located at the lateral side of the ankle. The fourth photo (4) indicates a neoplastic wound at the site of metastasis after left breast amputation. The fifth photo (5) is a pressure ulcer on the third day of *Lucilia sericata* larvae therapy. The last photo (6) shows a deep pressure ulcer with black and slushy necrotic tissue located in the area of the ischial tuberosity.

The task of the respondents was to rank the photos in order from the most repulsive (disgusting) to the least. Each digit could only be entered once. Each photo was assigned an Arabic numeral and was shown in a random order. The time that the subjects assumed to look at the photos was not longer than 2 min.

### 2.4. Statistical Analysis

The data collected in this study were analyzed with the use of the IBM SPSS Statistics 21 for Windows. The acceptable probability for a type 1 error was set at 5.0%, or *p* = 0.05. In order to evaluate the variable distributions, descriptive statistics were applied. Normal tests of distributions were conducted with Kolmogorov–Smirnov normality tests. The Pearson chi-square test was performed to test differences between classes of variables. The Spearman rho rank correlation was used to assess relationships among quantitative variables.

## 3. Results

### 3.1. Characteristics of the Respondents

All respondents were authorized to treat wounds and perform wound treatment procedures. The mean age of the subjects was 38.39 years; the median was 38 years (Table 1). The median of professional seniority was 14.5 years. The majority (82.9%) of the respondents declared higher education (Master or Bachelor of Nursing). Practically all (98.6%) declared post-graduate education, including at least one wound treatment course (65.7%) and nursing specialization (55.7%).

### 3.2. Perception of Wounds and Selected Variables

When subjects were asked to rank the six wound photographs “from most hideous to least hideous”, the photograph most commonly ranked as most hideous (disgusting) was photo #5, which shows a pressure ulcer wound containing larvae (Figure 1).

Since both women and men most often ranked photo #5 of the larvae feeding in the wound as the most hideous, a list of selected variables was made with the assumption that the sight of larvae in the wound is a visually unpleasant experience. The lack of a statistically significant difference was confirmed by the chi-square test (chi-square = 0.057, df = 1, *p* = 0.811). However, there was clearly a gender difference in the perceived repulsiveness between photograph #2 (necrotic foot wound in a geriatric patient at the end of life) and #4 (metastatic cancer wound within the amputated breast). Women much more often perceived photograph #2 as the second most repulsive photo (chi-square = 7.479, df = 1, *p* = 0.006), while men most often considered photo #4 to be the second most repulsive photo (chi-square = 8.102, df = 1, *p* = 0.004).

Level of work experience in the nursing profession also correlated with the perception of wound images. Photograph #5 was more often perceived as disgusting by nurses with the least number of years (up to five years) working in the profession (see Table 2). Comparing responses according to level of educational degree, photograph #5 was selected as the most disgusting by people with bachelor’s degrees in nursing more often than those with master’s degrees in nursing (see Table 3). Since very few subjects fell outside these two nursing degree groups, those few were not included in this analysis (one group consisted of eight nurses with highly specialized school degrees, while another comprised four with a PhD degree; however, the sizes of the groups were too small to compare them with other categories).

### 3.3. Perception of Wound Images and Readiness to Undertake MDT

**Figure 2:**
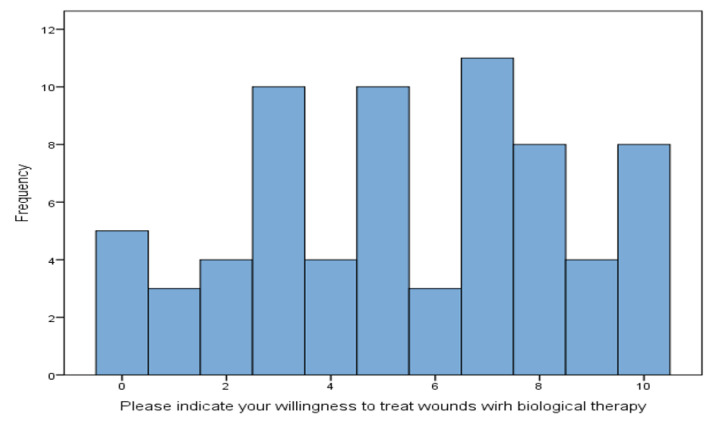
Self-assessment on willingness to undertaking biodebridement on 0–10 scale, where 0 = not ready, 10 = high readiness.

Subjects were asked to describe their readiness to undertake MDT (Figure 2) by selecting a corresponding number on a scale from 0 (not ready) to 10 (high readiness). The mean rating was 5.46 (SD = 3.001), however, with lack of normal distribution (Kolmogorov–Smirnov test, *p* = 0.002).

In order to assess the relationship between the self-assessed readiness to undertake MDT and the perception of wound images, the respondents’ answers were grouped into three categories of self-assessed readiness: low (31.4%), medium (40.0%), and high (28.6%). This division into three categories was based on the analysis of the original study population (290 subjects), after converting the scale to standard ten (sten) form: low scale values = 1–4 sten, medium = 5 and 6 sten, and high = 7–10 sten [18]. Those who declared a low self-assessed readiness to use biological therapy more often indicated photo No. 5 as the most disgusting compared to subjects declaring an average or high self-assessment score of readiness (Table 4). The difference was statistically significant.

**Figure 3:**
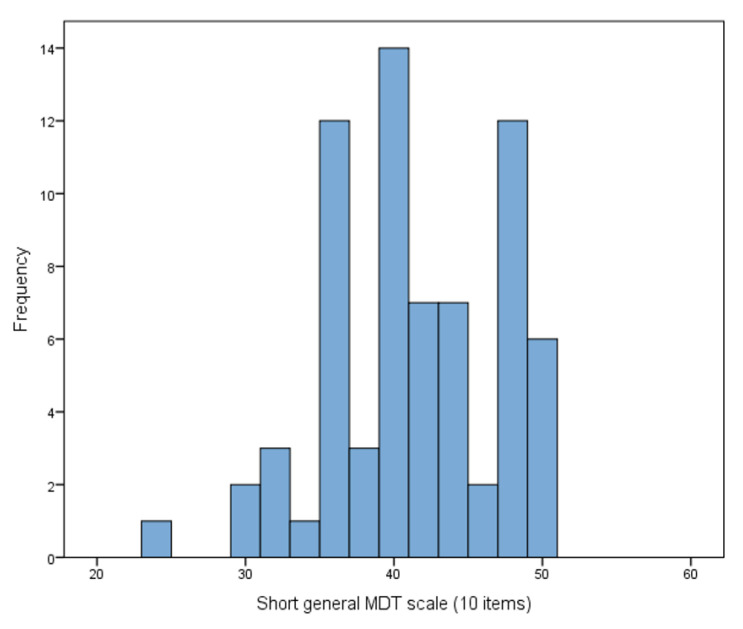
Readiness to undertake biodebridement as assessed with MDT 10.

Readiness to implement MDT was also evaluated as a function of the MDT 10 score (Figure 3). For the analysis, the MDT 10 score was transformed into three categories referring to the intensity of the feature: low (27.1%), medium (41.4%), and high (31.4%) (Table 5). The observed gender differences in the frequency of low and high MDT 10 scores were not statistically significant.

### 3.4. Perceived Stress and the Perception of Wound Images

Over 90% of subjects rated their stress level as medium or high; only 8.6% described their stress level as low. Because there were so few subjects in the “low stress” category, it was not valid to assess differences in photo selection on the basis of this stress category. Nevertheless, whether the association of stress level was assessed without including the “low stress” category or whether it was assessed by combining the low and medium stress categories together, the results of the chi-square test indicated that the choice of images (Table 6) and readiness to implement maggot therapy were not likely correlated with the perceived level of stress.

## 4. Discussion

Over the past two decades, interest in MDT has increased due to the indisputable scientific evidence confirming the effectiveness of larvae and their secretions in the processes of debridement, disinfection, and stimulation of local wound healing [8,10,12,13]. Experts from the World Health Organization (WHO) urgently call for a change in strategies for the ordination and use of antibiotics limited to clinical indications supported by tests and antibiograms. Thus, systemic targeted antibiotic therapy should be selected on the basis of the result of microbiological examination and knowledge of the penetration of the administered antibiotic into the skin and subcutaneous tissue. The selection of substances should take into account the range of action in accordance with the identification of microorganisms and resistance testing. Even if new drugs are developed without changing behavior, antibiotic resistance will still remain one of the main threats in medicine [25]. The implementation of MDT into clinical practice (2004) provides opportunities not only for wound debridement but also for the elimination of many microorganisms, including those that are multidrug-resistant [12,15,26]. However, despite scientific reports documenting the efficacy and safety of MDT, the adoption of this therapy into common clinical practice has been slow [8,10].

It was hypothesized that certain factors (gender, professional experience, stress level) may determine reluctance to introduce larval therapy into clinical practice. Studies to date have supported the ideas, at least to some degree, that disgust (the “yuck factor”) and ignorance about maggot therapy are two factors that impede acceptance of MDT [21,27,28]. The disgust and repulsion caused by maggots are often rooted in associations, negative experiences, and images encoded in childhood related to decomposing corpses, animal excrement, and rotting garbage. For the medical practitioner and the patient, the sight of larvae wriggling in the wound can trigger unpleasant visual and tactile experiences, the intensity of which can inhibit the implementation of the method [29,30]. Repulsive images and smells can stimulate the imagination and generate negative psychosomatic symptoms [27,31]. Furthermore, deficits in knowledge and skills concerning MDT, as well as lack of experience and institutional support, may inhibit the use of maggot therapy. Nurses are the largest group of medical professionals who prevent and treat wounds, especially in the outpatient setting. The appearance, movement, and odor associated with the larvae may be unpleasant, thereby evoking disgust and reluctance to utilize MDT. Some nurses describe their initial experiences with MDT therapy as being stressful, accompanied by sleep disturbances, ruminations, and visions of maggots wriggling in the wound [12,14]. Przybek-Mita et al. [16] found that the level of this stress may reduce the readiness to implement this modality in therapeutic protocols. Other studies have also suggested that gender may play a role in the acceptance of maggot therapy [21,27], although these differences did not reach statistical significance. The present study was undertaken to better assess whether the gender and variables such as professional experience and the level of stress of medical staff can really determine the perception and acceptance of MDT in clinical practice. This question was addressed by carefully examining a randomly assigned age-matched male-female paired subgroup of 70 nurses from the population of Rzeszow nurse trainees previously well described. In addition to gender, their demographic, educational, emotional, and attitudinal data were also compared and analyzed in order to consider other factors that might affect therapists’ level of disgust towards maggots and their degree of readiness to implement maggot therapy for wound care.

The analysis of subjects’ PSS10 scores, MDT 10 responses, and ranking of most disgusting images indicated that gender was not strongly correlated with a reluctance to implement MDT. In fact, only the range of professional work experience and knowledge about maggot therapy were significantly correlated with the level of disgust and willingness to use maggot therapy. Disgust over the maggots and less readiness to use maggot therapy was more often seen in nurses with less professional work experience and less knowledge about the treatment itself, regardless of gender. Greater comfort with maggots and maggot therapy were more often seen in nurses with more professional work experience and knowledge about maggot therapy, regardless of work experience or gender.

These findings build upon the results of earlier studies [16,21,27,28], especially defining the insignificant role of gender in influencing therapist attitudes towards maggot therapy and supporting the association of knowledge and wound care experience with greater comfort about maggots and maggot therapy.

Advanced nursing practice can improve and strengthen the health care system by maintaining a high standard of service and reducing the financial costs associated with treatment delays and complications. Nurses are responsible for implementing wound care prevention and intervention strategies for inpatients and outpatients [16,32]. Optimal nursing care is dependent on continued education, thoughtful practice, and the judicious use of professional autonomy [33,34]. In a holistic approach, the process of patient care is directed at the patient’s holistic assessment and psychosocial functioning in addition to wound care. Implementing prehabilitation guidelines, improving physical activity, and participating in the treatment of coexisting chronic diseases are among the key activities of complementary care. Reducing pain, improving dietary nutrition, and increasing physical activity can improve the patient’s subjective quality of life [35,36].

Education and experience reduce stress, not only in regards to maggot therapy but also in most endeavors. The greatest willingness to use MDT was seen in subjects with the lowest level of stress, even though the level of stress was not strongly associated with the visual and visceral responses to the wound images. The ideal teachers and mentors are those with knowledge and practical experience. Competent training may not eliminate stress completely, but when combined with motivation and good coping skills, stress can be reduced substantially and the quality of both service and life will be optimized [33]. The advancement of medical and health sciences is led by leaders who are infused with the spirit of mentorship. Mentorship itself is perceived as a certain human development strategy based on the relationship between a person with extensive experience in a certain field and a person who wants to gain that experience. This relationship is based on strong leadership and inspiration in addition to dialogue geared towards motivating the mentee to achieve specific goals. High therapeutic benefits and professional implementation of nursing students in wound care show that mentoring contributes to the development of clinical nursing and should therefore be utilized in the training and implementation of MDT in clinical practice [37,38].

Interest in MDT is constantly increasing due to the indisputable scientific evidence confirming the effectiveness of larval defensins in the process of debridement and stimulation of local wound healing. Many studies and meta-analyses confirm the benefits and efficacy of *Lucilia sericata* larvae in the treatment of chronic wounds [14,15,16,30,39,40]. As these types of wounds become more common and more problematic, MDT can play a key role in the local wound treatment process, especially for wounds related to diabetes and pressure injuries. Healthcare professionals—particularly nursing staff—are a key link in implementing, advocating, and administering wound care services and are integral to the smooth adoption of MDT into any wound care program. Showing the advantages of MDT and educating patients on its efficacy can be beneficial in resolving misconceptions about this form of therapy. This study, along with a growing number of related studies, indicates that maggot therapy education and experience must become a well-represented part of the nursing curriculum. Every effort should be made to investigate the feasibility of implementing larval therapy.

### Limitations

There are several factors that may limit the validity of applying these results to the general nursing community. First of all, the study was small and specific, comprising 290 Polish wound care nurses who volunteered to complete intensive evaluation and completed a series of questionnaires at the Postgraduate Education of Nurses and Midwives in Rzeszów, Poland [16,21]. Even though the present analysis has sampled a large percentage of this population (35 men and an age-matched pairing of 35 women, or a total sample size of 24%), these results may not completely extrapolate to other nursing or wound therapist populations. Therefore, similar studies should be repeated with different populations. The study did not evaluate whether or not women, in general, have more negative attitudes towards maggots; it only evaluated whether any such attitudes existed in female wound care nurses compared to male nurses and whether such attitudes inhibited their readiness to implement maggot therapy. Another limitation is that the present study focused only on selected variables (age, education, seniority in the profession, gender) that have a potential impact on the level of perceived stress and its impact on the readiness to implement MDT into practice; many other variables were not evaluated.

## 5. Conclusions

The use of medicinal maggots in the treatment of chronic wounds takes on a new meaning and urgency in the face of increasing bacterial resistance. Therefore, it is important to understand and overcome the mechanisms that impede the general acceptance of this modality. In contrast to some opinions, this analysis indicated that therapist’s perception of MDT was neither associated with their gender nor their perceived level of stress. Little professional experience in nursing and a lack of knowledge about the method (which were weakly correlated, Spearman’s rho = 0.207, *p* = 0.086), however, were associated with reluctance to use medicinal larvae in therapeutic protocols. In the process of educating medical personnel, it is also necessary to take measures related to the promotion of the method among patients with wounds. Educational activities should be undertaken to train therapists in the use of MDT in order to create positive attitudes and comfort with this useful wound care technique. These topics should be a part of nursing curricula at all levels of diploma and postgraduate education.

## Figures and Tables

**Table 1:** Demographic characteristics of the respondents.

		Males (N)	Females (N)	Total (N)	% of This Demographic
Total Subjects		35	35	70	100.0%
Age	24–34	15	15	30	42.9%
	35–44	14	14	28	40.0%
	45–54	4	4	8	11.4%
	55–64	2	2	4	5.7%
Age	Mean (SD)	38.43 (8.441)	38.34 (7.784)	38.39 (8.060)	
	Median (Range)	38.00 (24–57)	37.00 (26–57)	38.00 (24–57)	
Education	Registered nurse	4	4	8	11.4%
	Bachelor of nursing	7	7	14	20.0%
	Master of nursing	24	24	48	68.6%
Work experience in the profession of a nurse	1–5 years	6	6	12	17.1%
	6–10 years	6	6	12	17.1%
	11–15 years	9	9	18	25.7%
	16–20 years	6	6	12	17.1%
	21–30 years	6	6	12	17.1%
	More than 30 years	2	2	4	5.7%
Work experience (years)	Mean (SD)	15.37 (8.630)	13.63 (8.461)	14.50 (8.529)	
	Median (Range)	14.00 (1–35)	15.00 (2–36)	14.50 (1–36)	

**Table 2:** Selection of photo No. 5 as the most disgusting photo in categories of seniority in the nursing profession.

			Work Experience in the Profession of a Nurse				
		Total	1–5 yrs	6–10 yrs	11–15 yrs	16–20 yrs	≥21 yrs
Photo 5 chosen as the most disgusting	N	33	10	8	5	6	4
	%	47.1%	83.3%	66.7%	27.8%	50.0%	25.0%
Other photo chosen as the most disgusting	N	37	2	4	13	6	12
	%	52.9%	16.7%	33.3%	72.2%	50.0%	75.0%
Total	N	70	12	12	18	12	16
	%	100.0%	17.1%	17.1%	25.7%	17.1%	22.9%

chi-square = 14.039, df = 4, *p* = 0.007.

**Table 3:** Selection of photo No. 5 as the most disgusting photo in categories of education.

		Total	Bachelor in Nursing	Master in Nursing
Photo 5 chosen as the most disgusting	N	29	11	18
	%	50.0%	78.6%	40.9%
Other photo chosen as the most disgusting	N	29	3	26
	%	50.0%	21.4%	59.1%
Total	N	58	14	44
	%	100.0%	24.1%	75.9%

chi-square = 6.026, df = 1, *p* = 0.014.

**Table 4:** Selection of photo No. 5 as the most disgusting photo in categories of self-assessment of readiness to use biological therapy based on the MDT 10 questionnaire.

	Total	Self-Assessment of Readiness to Use Biological Therapy		
		Low (0–3)	Medium (4–7)	High (8–10)
Photo 5 selected as the most disgusting	33	16	10	7
	47.1%	72.7%	35.7%	35.0%
Other photo chosen as the most disgusting	37	6	18	13
	52.9%	27.3%	64.3%	65.0%
Total	70	22	28	20
	100.0%	31.4%	40.0%	28.6%

chi-square = 8.430, df = 2, *p* = 0.015.

**Table 5:** Readiness to undertake biodebridement as assessed with MDT10 in categories of gender.

Scale MDT (10 Items)	Total	Woman	Man
Low result	19	12	7
	27.1%	34.3%	20.0%
Medium result	29	14	15
	41.4%	40.0%	42.9%
High result	22	9	13
	31.4%	25.7%	37.1%
Total	70	35	35
	100.0%	50.0%	50.0%

chi-square = 2.078, df = 2, *p* = 0.354.

**Table 6:** Perception of wound images (frequency of a “most disgusting” rank) categorized by the level of stress intensity.

Frequency of a “Most Disgusting” Rank	Total	Stress Intensity Level		
		Low Stress Level	Average Stress Level	High Stress Level
Photo 1	0	0	0	0
	0.0%	0.0%	0.0%	0.0%
Photo 2	18	2	9	7
	25.7%	33.3%	23.7%	26.9%
Photo 3	0	0	0	0
	0.0%	0.0%	0.0%	0.0%
Photo 4	16	3	5	8
	22.9%	50.0%	13.2%	30.8%
Photo 5	33	1	22	10
	47.1%	16.7%	57.9%	38.5%
Photo 6	3	0	2	1
	4.3%	0.0%	5.3%	3.8%
Total	70	6	38	26
	100.0%	8.6%	54.3%	37.1%

## Data Availability

The data presented in this study are available on reasonable request from the corresponding author: pwiech@ur.edu.pl.
